# Stigma and quality of co‐located care for HIV‐positive people in addiction treatment in Ukraine: a cross‐sectional study

**DOI:** 10.1002/jia2.25492

**Published:** 2020-05-06

**Authors:** Yuliia Sereda, Tetiana Kiriazova, Olena Makarenko, Jennifer J Carroll, Natasha Rybak, Andriy Chybisov, Sally Bendiks, Bulat Idrisov, Arunima Dutta, Fizza S Gillani, Jeffrey H Samet, Timothy Flanigan, Karsten Lunze

**Affiliations:** ^1^ Ukrainian Institute on Public Health Policy Consultant Kyiv Ukraine; ^2^ Ukrainian Institute on Public Health Policy Kyiv Ukraine; ^3^ Elon University Elon NC USA; ^4^ Warren Alpert Medical School of Brown University Providence RI USA; ^5^ American Cancer Society Washington DC USA; ^6^ Boston Medical Center Boston University Boston MA USA; ^7^ Bashkir State Medical University Ufa Russia; ^8^ Federal Research Institute for Health Organization and Informatics of Ministry of Health of the Russian Federation Moscow Russia; ^9^ Moscow Institute of Physics and Technology Moscow Russia

**Keywords:** discrimination, bias, HIV, injection drug use, quality of care, co‐located care

## Abstract

**Introduction:**

Co‐located treatment for HIV and opioid use disorder has been shown to improve care outcomes for HIV‐positive people who inject drugs (PWID) in Ukraine. However, patients continue to be stigmatized for both HIV and substance use. This study aimed to assess whether co‐located care for HIV‐positive PWID receiving opioid agonist treatment (OAT) services in Ukraine is associated with less stigma and better perceived quality of HIV services.

**Methods:**

This cross‐sectional study enrolled 191 HIV‐positive PWID who received OAT services at three healthcare facilities providing substance use treatment (OAT only) and at four facilities that provided co‐located care (both OAT and HIV treatment) in six regions in Ukraine during July‐September, 2017. Primary outcomes were HIV stigma (Berger scale), substance use stigma (Substance Abuse Stigma Scale) and intersectional stigma (both stigma forms above 75th percentile). Secondary outcome was quality of HIV care, a composite score based on a package of received services. Linear and ordinal regressions were used to assess the predictors of selected outcomes.

**Results:**

Study participants were 75% male, mean age 40 ± 7 years; 47% received co‐located care, and 10.5% had both high HIV and substance use stigma. Co‐located care was neither associated with HIV nor substance use stigma but it was linked to better quality of HIV care (adjusted odds ratio: 4.13; 95% CI: 2.31, 7.54). HIV stigma was associated with suicide attempts (adjusted beta (aβ): 5.90; 95% CI: 2.05, 9.75), and substance use stigma was linked to poor mental health (aβ: −0.26; 95% CI: −0.44, −0.08) and lower likelihood of receipt of services from non‐governmental organization (NGO; aβ: −6.40; 95% CI: −10.23, −2.57).

**Conclusion:**

One in ten people with HIV in this cohort who received OAT services experienced high levels of both HIV and substance use stigma, which was associated with poorer mental health and less NGO support. Co‐located HIV and OAT services were linked to better perceived quality of HIV care, but did not seem to reduce stigma for this key population. Stigma interventions for PWID, possibly delivered involving NGOs, may be an approach to mitigate this challenge.

## 1 Introduction

Stigma, the social exclusion of individuals labelled with an undesirable trait, remains one of the key barriers discouraging people from seeking and receiving services for HIV and associated diseases [1]. About one in eight people living with HIV (PLHIV) reported being denied health services because of stigma and discrimination [2]. HIV‐related stigma has been linked to numerous poor health outcomes: negative effects on mental health [3, 4, 5]; unhealthy alcohol use [3, 6, 7]; poor antiretroviral treatment (ART) adherence [3, 8]; and lower overall quality of life [3, 9]. In addition to HIV‐related stigma, key populations are also affected by stigma relating to people with other identities and behaviours, such as people who use substances [10], people engaged in sex work [11], sexual minorities [12] and people under 25 years of age [13].

Stigma research among PLHIV rarely utilized an intersectionality framework incorporating multiple levels of stigma in their study [14]. Intersectional stigma can results from the layering of multiple marginalized statuses [15]. The few intersectional stigma studies conducted on HIV and substance use found that HIV‐positive people who inject drugs (PWID) endorse internalized stigma (experienced or expected from others by affected people) and public stigma (created by public attitudes) related to substance use, in addition to HIV stigma; both forms were linked to poor mental health and less engagement in treatment [16, 17].

PWID are a highly marginalized and stigmatized group, and even more so are those living with HIV [16, 17, 18, 19]. Due to stigma, health providers may perceive patients with substance use disorders as problematic and therefore be less motivated to initiate their HIV treatment, an assumption shown to be unfounded [17, 19].

In response to the high prevalence of HIV among PWID in Ukraine (23%) [20, 21], some healthcare facilities have started offering co‐located HIV and opioid agonist treatment (OAT) for opioid use disorders. Provision of HIV, OAT, and other health services at one location can increase access to services for HIV‐positive PWID by improving accessibility, user‐friendliness, and comprehensiveness of care [20, 21, 22, 23]. However, negative attitudes about methadone or buprenorphine (OAT medications) can stigmatize patients using OAT services [24, 25, 26]. Notably, little is known about the effect of care co‐location on patients’ perceived stigma. In particular, it is unknown whether co‐located care reduces patients’ perception or expectation of HIV and substance use stigma.

This study aimed to assess whether HIV and substance use treatment co‐location for HIV‐positive PWID receiving OAT services in Ukraine is associated with lower stigma levels, compared to OAT only sites. We also explored association between treatment co‐location and perceived quality of care. We hypothesized that co‐located care is associated with lower stigma levels and better quality of care, compared to OAT only sites.

## 2 Methods

### 2.1 Study design

We conducted a cross‐sectional study at seven healthcare facilities in six regions of Ukraine. We collected data at three sites providing OAT only (two branches of narcological clinic in Kyiv and narcological dispensary in Mykolaiv) and at four sites that provided co‐located OAT and HIV treatment services (narcological dispensaries in Dnipro, Lviv and Cherkasy and AIDS Center in Odesa).

### 2.2 Country settings

Ukraine has the second‐largest HIV epidemic in Eastern Europe and Central Asia [20, 21]. HIV prevalence is the highest among PWID (23%) followed by males who have sex with males (7.5%) and sex workers (5%) [20, 21].

In 2017, the estimated PWID population in Ukraine was 350,300 with over 80,600 of them being HIV‐positive [20, 21]. Among HIV‐positive PWID, 58% are aware of their status, and 38% receive ART [20, 21]. This epidemiologic situation is further complicated by the reality that tuberculosis (TB) coinfection is very common in Ukraine [27].

### 2.3 Study settings

In Ukraine, OAT is delivered through a network of governmental healthcare facilities and is not available in prisons [28]. Patients receive OAT at narcological dispensaries (46%), special sites in hospitals (31%), primary care centres (6%), HIV clinics (6%), TB clinics (6%) and mental health clinics (5%) [29]. In 2018, an estimated 3% (11,385 of 350,300) of the PWID population received OAT services in Ukraine; most of them (10,293 of 11,385; 90%) were on methadone treatment while other patients received buprenorphine [29]. The estimated OAT coverage among HIV‐positive PWID is 6% [29].

One study site (AIDS Center in Odesa) was originally an HIV clinic founded in 1999 that started to prescribe OAT in 2014. Other study sites were substance use treatment clinics which introduced OAT between 2008 and 2012 (narcological clinics in Cherkasy, Dnipro, Kyiv, Lviv and Mykolaiv). During 2012‐2015, HIV treatment sites were created at narcological clinics in Cherkasy, Dnipro and Lviv.

In Ukraine, co‐located OAT and HIV care is delivered by two distinct service providers, i.e. at narcological clinics (OAT sites) and at HIV clinics (ART sites), that are located in the same building. Providers at co‐located care sites routinely communicate to discuss patient care, while providers at OAT sites without co‐located HIV services usually do not communicate with HIV specialists.

HIV testing is available at both narcological and HIV clinics. HIV‐positive PWID identified at narcological clinics are referred to HIV clinics for diagnosis, ART prescription and monitoring. PWID identified at HIV clinics are referred to narcological clinics for addiction diagnosis and treatment, including OAT. OAT is supervised by Addiction Treatment Specialists, while HIV treatment is supervised by Infectiouos Disease Specialists.

Only 20% of OAT patients in Ukraine are on self‐administered treatment and receive take‐home doses for 10 days maximum [29]. Most patients visit OAT sites every day and receive methadone or buprenorphine under direct supervision of a health worker. Patients may be allowed to take OAT at home if they have proven compliance with treatment during six months [30]. OAT treatment monitoring, including urine tests for the presence of opioids, is conducted at least once per three months [30]. Frequency of visits is different for HIV treatment. Typically, HIV patients receive take‐home ART doses for 30 days immediately after treatment initiation, and this period may be extended up to 90 days in case of consistent treatment compliance [31]. National HIV treatment guidelines recommend at least one visit per six months for HIV treatment monitoring (i.e. CD4 and viral load testing) [31].

According to the national legislation, OAT and ART services can be provided only by physicians and nurses, and therefore they are not available in non‐governmental organizations (NGOs) working with PWID. NGOs provide case‐management, social support and counselling, information and education, condom distribution and sterile syringes programmes. Four of the seven selected sites (Cherkasy, Dnipro, Mykolaiv, and Odesa) had a co‐located NGO providing services to PWID at the healthcare facility.

### 2.4 Participants

Eligibility criteria for the study participants were the following: age ≥18 years; lifetime history of drug injection (by self‐report); HIV‐positive status (by self‐report); receiving OAT; fluent in Russian or Ukrainian. The exclusion criterion was cognitive impairment resulting in inability to provide informed consent based on an assessment during the consent process.

### 2.5 Sample

We recruited a consecutive sample of HIV‐positive individuals receiving OAT, who were referred to the study by their healthcare providers. In total, 198 participants were screened for the study. Of them, 191 met inclusion criteria, participated in the study and were included in the data analysis.

### 2.6 Study procedures and period

The Ukrainian Institute on Public Health Policy collected data from July through September 2017. An on‐site research assessor screened patients referred by their healthcare provider at the clinic for eligibility, offered eligible individuals participation in the study, obtained informed consent, and administered the survey in a private location at the clinic. Survey measures were pilot‐tested and administered in Russian or Ukrainian, as to the participant’s preference. All study staff was trained on the study protocol, human subjects protection and participant assessment prior to recruitment.

We collected all study data via Research Electronic Data Capture tools [32]. Participants received 200 Ukrainian Hryvnia (the equivalent of US $8 at the time of study) in cash as compensation for their time and transportation expenses.

### 2.7 Variables

Primary outcomes included HIV and substance use stigma scores, and intersectional stigma (i.e. those who reported high scores for both stigma forms). We measured HIV stigma with the abbreviated Berger Scale [33]. Substance use stigma was assessed with an abbreviated Substance Abuse Stigma Scale [34]. Minimum‐maximum standardization with a range from 0 to 100 was used to make total HIV and substance use stigma scales comparable. The intersectional stigma variable was categorized as “both HIV and substance use stigma score high”; “high HIV stigma only,” “high substance use stigma only,” “medium or low scores for both HIV and substance use stigma.” Scores ≥75th percentile were considered as high stigma level.

Secondary outcome was quality of HIV care measured as HIV Care Quality Index (HCQI) [22]. The scale includes four items related to coverage of HIV services recommended per national guidelines at the time of study (at least one regular HIV check‐up within the last six months; at least one CD4 test within the last six months; receipt of ART; undetectable viral load); two items related to TB services (at least one TB screening within the last 12 months; receipt of isoniazid preventive therapy for TB); two items related to OAT (receipt of World Health Organization recommended dosage of ≥80mg of methadone or ≥12mg of buprenorphine; and no injection drug use during last 30 days); and two additional items on the receipt of hepatitis C virus (HCV) screening and receipt of referrals for other services or support. The HCQI scores ranged from 0 to 100, indicating the percentage met of the ten indicators for care among HIV‐positive OAT patients.

Covariates included demographic, behavioural and clinical characteristics of the study participants as well as perceptions on health care services and social support. Demographic variables were age, gender, marital status, household size, education, employment, personal monthly income in the national currency (Ukrainian Hryvnias) dichotomized at median, and history of incarceration. Behavioural variables included illicit drug use during the past 30 days, number of sexual partners in the last year, condomless sex in the last year, history of selling sex, smoking and unhealthy alcohol use per Alcohol Use Disorders Identification Test, short version (AUDIT‐C) score [35]. Clinical characteristics were time since first HIV‐positive test, CD4 cell count, history of TB and HCV infection, perceived mental and physical health measured by short form health survey version 2.0 (SF‐12v2, higher scores indicating better health) [36], past suicide attempts, OAT drug (methadone or buprenorphine) and duration. All clinical variables were based on self‐report. Accessibility and user‐friendliness scales were constructed based on the approach used in a previous Ukrainian study of client satisfaction with HIV services [37]. Social support scale was measured using modified version of the Duke University‐University of North Carolina Functional Support Questionnaire [38].

We pilot tested the questions for the main variables among five participants at one site in Kyiv prior to the study. To assess internal consistency of the main variables, we conducted confirmatory factor analysis and calculated Cronbach's alphas (α) for the following scales: (1) HIV stigma (11 items and 4 latent factors: α = 0.75; comparative fit index (CFI) = 0.969, Tucker‐Lewis index (TLI) = 0.955, standardized root mean square residual (SRMR) = 0.089); (2) substance use stigma (21 items and 4 latent factors: α = 0.83; CFI = 0.925, TLI = 0.914, SRMR = 0.100); (3) HCQI (10 items and 1 latent factor: α = 0.54; CFI = 0.945, TLI = 0.926, SRMR = 0.114); (4) social support scale (10 items and 1 latent factor: α = 0.89; CFI = 0.978, TLI = 0.971, SRMR = 0.099); (5) user‐friendliness scale (8 items and 1 latent factor: α = 0.88; CFI = 1.000, TLI = 1.000, SRMR = 0.039); (6) accessibility scale (5 items and 1 latent factor: α = 0.68; CFI = 0.990, TLI = 0.980, SRMR = 0.076); (7) SF‐12v12 scale (12 items and 2 latent factors: α = 0.89; CFI = 0.973, TLI = 0.966, SRMR = 0.091). Applying thresholds of α > 0.7 for satisfactory internal consistency of scales, CFI, TLI > 0.9 and SRMR < 0.1 for factor validity, we found all items except HCQI sufficiently valid and internally consistent. We used the HCQI for comparison with a previous study conducted in Ukraine [22].

### 2.8 Data analysis

Characteristics of the study participants stratified by the type of site were summarized with descriptive statistics and compared by chi‐squared tests for categorical variables, T‐tests for continuous variables with approximately normal distribution and Kruskal‐Wallis tests for continuous variables with skewed distribution. HIV and substance use stigma scores were normally distributed, and we assessed their predictors using linear regression. We estimated Intersection between HIV and substance use stigma, depicted as number of people with a given substance use stigma score quartile within each HIV stigma quartile. The HCQI score distribution was right‐skewed. To assess the HCQI predictors, the score was categorized by quartiles (<70, 70‐79, 80‐89, ≥90 out of 100 points), and ordinal regression analysis was applied. Multivariable regressions for stigma outcomes and HCQI included variables that were statistically significantly (*p* < 0.05) in simple regressions with one predictor in addition to exposure of interest (co‐located care for stigma outcomes, co‐located care and intersectional stigma for HCQI). Presence of multicollinearity was tested with the help of variance inflation factor (VIF). A VIF of 10 was considered as evidence of collinearity. Missing data were excluded from the analysis in the following variables: number of sexual partners (n = 12), history of selling sex (n = 1), smoking (n = 1), AUDIT‐C (n = 1), history of TB (n = 1), diagnosed with HCV (n = 9), history of suicide (n = 1), OAT drug (n = 1), receiving regular HIV check‐up (n = 1), CD4 testing (n = 7), receipt of HCV screening (n = 1), receipt of referrals (n = 1), HIV stigma (n = 1) and substance use stigma (n = 1). We conducted all analyses in R version 3.5.2 (The R Foundation for Statistical Computing, Vienna, Austria).

### 2.9 Ethics

The study protocol and instruments received ethical approval from the Institutional Review Boards at Boston University Medical Campus, The Miriam Hospital, and the Ukrainian Institute on Public Health Policy. All study participants provided informed consent.

## 3 Results

### 3.1 Characteristics of the study participants

Of the 191 participants, close to half (47%) were recruited at healthcare facilities with co‐located care (Table 1). The sample was predominantly male (75%), with a mean age of 40 years (SD: 7 years).

**Table 1:** Socio‐demographic, behaviour and clinical characteristics of the study participants stratified by the type of healthcare facility at seven sites in Ukraine, n = 191

	Total n (%)	Co‐located OAT and ART sites n (%)	OAT only sites n (%)	*p*
Total	191 (100)	90 (47)	101 (53)	
Demographical characteristics				
Mean age, years (SD, min‐max)	40 (7, 25‐59)	40 (8, 25‐59)	40 (6, 30‐57)	0.98
Female gender	48 (25)	24 (27)	24 (24)	0.64
Married/have a regular partner	80 (42)	37 (41)	43 (43)	0.88
Median household size, persons (Q1‐Q3; min‐max)	3 (2‐4; 1‐9)	2 (2‐3; 1‐9)	3 (2‐4; 1‐8)	0.23
Living with kids (under 18 years)	58 (30)	22 (24)	36 (36)	0.09
Education				
High school or less	75 (39)	34 (38)	41 (40)	0.88
Vocational	77 (40)	38 (42)	39 (39)	
Higher (academic degree)	39 (21)	18 (20)	21 (21)	
Employment				
Full or part‐time employment	113 (59)	54 (60)	59 (58)	0.82
Unemployed (including housewives and students)	78 (41)	36 (40)	42 (42)	
Median personal monthly income, UAH (1U.S. dollar = 26 UAH) (Q1‐Q3; min‐max)	1667 (1200‐3000; 0‐10,000)	1500 (1167‐2875; 300‐10,000)	1667 (1250‐3000; 0‐10,000)	0.47
History of incarceration	129 (68)	64 (71)	65 (64)	0.32
Behavioural characteristics				
Any illicit drug use during the last 30 days	87 (46)	34 (38)	53 (53)	0.042
Number of sexual partners during the last 12 months (anal/vaginal sex)^a^				
No sexual partners	42 (24)	22 (26)	20 (22)	0.77
1 sexual partner	103 (58)	49 (57)	54 (58)	
≥2 sexual partners	34 (19)	15 (17)	19 (20)	
Condomless sex during the last 12 months (anal/vaginal)	73 (38)	41 (46)	32 (32)	0.049
History of selling sex^a^	14 (7)	8 (9)	6 (6)	0.45
Everyday or occasional smoker	179 (94)	80 (89)	99 (99)	0.003
Median AUDIT‐C score (Q1‐Q3; min‐max)^a^	1 (0‐3; 0‐12)	1 (0‐3; 0‐10)	2 (1‐4; 0‐12)	0.033
Unhealthy alcohol use^a^ (AUDIT‐C ≥ 3 for females and ≥ 4 for males)	46 (24)	16 (18)	30 (30)	0.05
Clinical characteristics				
Median time since first HIV‐positive test result, years (Q1‐Q3; min‐max)	8 (3‐13; 0‐23)	8 (3‐13; 0‐23)	8.5 (4‐14; 0‐21)	0.87
Median CD4 count (Q1‐Q3; min‐max)	400 (251‐540; 20‐1970)	400 (265‐575; 20‐1000)	398 (251‐520; 22‐1970)	0.87
History of TB^a^	45 (24)	18 (20)	27 (27)	0.26
Diagnosed with HCV^a^	160 (88)	71 (87)	89 (90)	0.49
Mean SF12v2 physical health T‐score (SD, min‐max) (min = 18, max = 59)	41 (9, 18‐59)	41 (9, 18‐58)	41 (10, 18‐59)	0.66
Mean SF12v2 mental health T‐score (SD, min‐max)	38 (10, 13‐60)	39 (10, 16‐60)	37 (9, 13‐59)	0.11
Ever attempted suicide^a^	54 (28)	18 (20)	36 (36)	0.015
OAT drug^a^				
Methadone	147 (77)	83 (92)	64 (64)	<0.001
Buprenorphine	43 (23)	7 (8)	36 (36)	
Median OAT duration, years (Q1‐Q3; min‐max)	2 (0‐4; 0‐12)	1 (0‐3; 0‐8)	3 (1‐4; 0‐12)	<0.001
Attitudes				
Mean perceived accessibility of the healthcare facility (SD, min‐max)	76 (22, 7‐100)	83 (18, 33‐100)	70 (24, 7‐100)	0.002
Mean perceived user‐friendliness of the healthcare facility (SD, min‐max)	73 (24, 13‐100)	79 (21, 25‐100)	68 (26, 13‐100)	0.029
Mean perceived social support score (SD, min‐max)	73 (22, 0‐100)	75 (23, 0‐100)	71 (21, 10‐100)	0.11
Indicators for quality of HIV care				
Visited physician for a regular HIV check‐up within the last six months^a^	165 (87)	82 (91)	83 (83)	0.10
Tested for CD4 count within the last six months^a^	164 (90)	78 (91)	86 (89)	0.65
Currently on ART	156 (82)	81 (90)	75 (74)	0.005
Reached undetectable HIV viral load	94 (49)	53 (59)	41 (41)	0.012
Received screening for TB during last 12 months	188 (98)	88 (98)	100 (99)	0.49
Ever received Isoniazid preventive therapy for tuberculosis	115 (60)	69 (77)	46 (46)	<0.001
Receives WHO‐recommended OAT dosing (methadone ≥ 80mg or buprenorphine ≥ 12mg)	117 (61)	57 (63)	60 (59)	0.58
Injecting drug use during last 30 days	47 (25)	20 (22)	27 (27)	0.47
Received HCV screening^a^	181 (95)	82 (91)	99 (99)	0.011
Received referrals for additional services or support^a^	126 (66)	70 (78)	56 (56)	0.002
Median HIV Care Quality Index (Q1‐Q3, min‐max)	80 (70‐90; 10‐100)	90 (70‐90; 30‐100)	80 (60‐80; 10‐100)	<0.001
Stigma scores				
Mean HIV stigma score (SD, min‐max)^a^	49 (12, 20‐87)	49 (12, 27‐87)	49 (12, 20‐83)	0.88
Mean substance use stigma score (SD, min‐max)^a^	53 (13, 23‐86)	53 (12, 24‐82)	53 (14, 23‐85)	0.62

Data was summarized as n (%) unless other stated.

ART, antiretroviral treatment; AUDIT‐C, The Alcohol Use Disorders Identification Test, short version; HCV, hepatitis C virus; OAT, opioid agonist treatment; Q1, 25th percentile; Q2, 75th percentile; SD, standard deviation; SF12v2, Short Form Health Survey questionnaire, version 2; TB, tuberculosis; UAH, Ukrainian Hryvnias; WHO, World Health Organization.

^a^ Missing data was excluded for next variables: number of sexual partners (n = 12), history of selling sex (n = 1), smoking (n = 1), AUDIT‐C (n = 1), history of TB (n = 1), diagnosed with HCV (n = 9), history of suicide (n = 1), OAT drug (n = 1), receiving regular HIV check‐up (n = 1), CD4 testing (n = 7), receipt of HCV screening (n = 1), receipt of referrals (n = 1), HIV stigma (n = 1) and substance use stigma (n = 1).

Patients at OAT only sites reported more substance use (53% compared to 38% at co‐located care) and more unhealthy alcohol use (30% vs. 18% at co‐located care), while condomless sex was more frequent among patients in co‐located care (46% compared to 32% at OAT only sites).

Most study participants (77%) received methadone, all the rest buprenorphine. On average, participants were satisfied with the health services they received and with their social support. Mean scores were 76 (SD: 22) out of 100 for accessibility; 73 (SD: 24) for user‐friendliness, and 73 (SD: 22) for social support.

### 3.2 HIV stigma

The mean HIV stigma score of 49 out of 100 (SD: 12) was identical at both co‐located OAT and ART sites and OAT only sites. In adjusted analyses, a history of suicide attempt was associated with greater HIV stigma (adjusted beta (aβ): 5.90; 95% CI: 2.05, 9.75, Table 2).

**Table 2:** Factors associated with separate HIV and substance use stigma scores among HIV‐positive PWID receiving OAT at seven sites in Ukraine: linear regression analysis, n = 190^a^

	HIV stigma score		Substance use stigma score	
	Crude beta (95% CI)	Adjusted beta (95% CI)	Crude beta (95% CI)	Adjusted beta (95% CI)
Co‐located OAT and ART sites (ref. OAT only sites)	0.25 (−3.16, 3.66)	1.55 (−1.73, 4.84)	−0.31 (−4.06, 3.43)	3.14 (−0.15, 6.43)
Demographical characteristics				
Age, years	0.02 (−0.23, 0.27)	–	−0.15 (−0.42, 0.12)	–
Male gender (ref. female)	−3.20 (−7.09, 0.69)	–	−1.47 (−5.76, 2.83)	–
Married/have regular partner (ref. single, never married, divorced, separated or widowed)	0.20 (−3.25, 3.65)	–	−3.38 (−7.13, 0.38)	–
Household size, persons	−0.09 (−1.11, 0.93)	–	0.52 (−0.60, 1.64)	–
Living with children under 18 years (ref. no)	1.26 (−2.43, 4.96)	–	1.01 (−3.05, 5.06)	–
High school or less (ref. higher education)	1.52 (−3.11, 6.16)	–	3.26 (−1.82, 8.33)	–
Vocational (ref. higher education)	−0.01 (−4.63, 4.62)	–	1.29 (−3.77, 6.35)	–
Unemployed including housewives and students (ref. full or part‐time employment)	1.82 (−1.64, 5.27)	–	**4.22 (0.47, 7.97)**	3.14 (−0.20, 6.47)
Personal monthly income above the median: >1667 UAH^b^ (ref. median or below)	**−3.50 (−6.88, −0.12)**	−2.51 (−5.85, 0.83)	−1.39 (−5.13, 2.36)	–
History of incarceration (ref. no)	−1.90 (−5.52, 1.72)	–	−1.92 (−5.89, 2.06)	–
Behavioural characteristics				
Any illicit drug use during the last 30 days (ref. no)	−1.17 (−4.58, 2.24)	–	3.26 (−0.46, 6.98)	–
1 sexual partner during the last 12 months (anal/vaginal sex) (ref. no partners)	−0.64 (−4.93, 3.65)	–	−1.00 (−5.74, 3.73)	–
≥2 sexual partners during the last 12 months (anal/vaginal sex) (ref. no partners)	1.47 (−3.94, 6.88)	–	2.76 (−3.21, 8.73)	–
Condomless sex during the last 12 months (anal/vaginal) (ref. no)	−0.44 (−3.94, 3.06)	–	−0.29 (−4.13, 3.55)	–
History of selling sex (ref. no)	0.35 (−6.16, 6.87)	–	0.84 (−6.31, 7.99)	–
Everyday or occasional smoker (ref. smoked before or never smoked)	−3.29 (−10.56, 3.99)	–	2.56 (−5.43, 10.55)	–
AUDIT‐C score	−0.43 (−1.11, 0.25)	–	0.40 (−0.35, 1.14)	–
Clinical characteristics				
Time since first HIV‐positive test result, years	−0.12 (−0.39, 0.15)	–	−0.09 (−0.39, 0.20)	–
CD4 count	0.00 (−0.01, 0.00)	–	0.00 (−0.01, 0.01)	–
History of TB (ref. no)	1.68 (−2.32, 5.68)	–	−0.63 (−5.03, 3.76)	–
Diagnosed with HCV (ref. no)	1.65 (−3.84, 7.14)	–	2.10 (−3.87, 8.07)	–
SF12v2 physical health T‐score	**−0.25 (−0.43, −0.07)**	−0.13 (−0.32, 0.05)	−0.17 (−0.38, 0.03)	–
SF12v2 mental health T‐score	**−0.27 (−0.44, −0.09)**	−0.15 (−0.34, 0.03)	**−0.46 (−0.65, −0.27)**	**−0.26 (−0.44, −0.08)**
Ever attempted suicide (ref. no)	**7.31 (3.68, 10.93)**	**5.90 (2.05, 9.75)**	**8.13 (4.15, 12.10)**	2.10 (−1.59, 5.79)
OAT drug: methadone (ref. Buprenorphine)	−1.86 (−5.92, 2.20)	–	−0.33 (−4.79, 4.14)	–
OAT duration, years	0.25 (−0.41, 0.91)	–	0.00 (−0.72, 0.72)	–
Attitudes				
Perceived accessibility of the healthcare facility	−0.02 (−0.10, 0.05)	–	**−0.10 (−0.19, −0.02)**	−0.05 (−0.12, 0.03)
Perceived user‐friendliness of the healthcare facility	−0.01 (−0.08, 0.06)	–	−0.06 (−0.13, 0.02)	–
Social support score	**−0.10 (−0.18, −0.02)**	−0.04 (−0.12, 0.04)	**−0.13 (−0.21, −0.04)**	−0.02 (−0.10, 0.06)
Receipt of services at non‐governmental organization (ref. no)	1.91 (−1.92, 5.74)	–	**−5.79 (−9.93, −1.67)**	**−6.40 (−10.23, −2.57)**

Values in bold indicate statistically significant associations.

ART, antiretroviral treatment; AUDIT‐C, The Alcohol Use Disorders Identification Test, short version; CI, confidence interval; HCV, hepatitis C virus; ref., reference category; OAT, opioid agonist treatment; PWID, people who inject drugs; SF12v2, Short Form Health Survey questionnaire, version 2; TB, tuberculosis; UAH, Ukrainian Hryvnias; WHO, World Health Organization.

^a^ Missing data on HIV and substance use stigma scores was excluded (n = 1).

^b^ 1U.S. dollar = 26 UAH.

### 3.3 Substance use stigma

Similarly to HIV stigma, substance use stigma scores did not differ at co‐located OAT and ART sites or OAT only facilities, with a mean score of 53 out of 100 (SD: 13). On average, when comparing standardized scales, the level of substance use stigma was higher among study participants compared to HIV stigma (mean difference: 3.91; 95% CI: 1.39, 6.42).

Substance use stigma scores were associated with worse mental health (aβ = −0.26; 95% CI: −0.44, −0.08). Participants who reported receipt of services from NGO had lower level of substance use stigma than those who do not (aβ = −6.40; 95% CI: −10.23, −2.57).

### 3.4 Intersectional stigma

**Figure 1:**
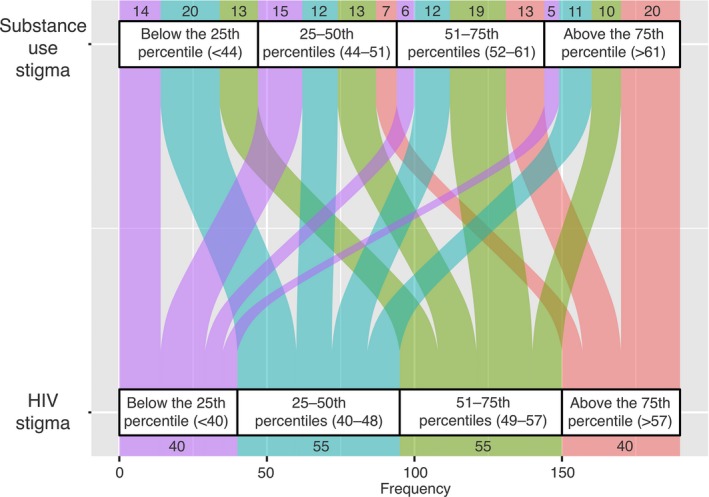
Intersection between HIV and substance use stigma scores among HIV‐positive PWID receiving opioid agonist therapy at seven sites in Ukraine, n = 190^†^. ^†^This chart shows the intersection between HIV and substance use stigma, depicted as number of people with a given HIV stigma score quartile within each substance use stigma quartile. Missing data on HIV and substance use stigma scores were excluded (n = 1). PWID, people who inject drugs.

HIV and substance use stigma scores moderately correlated with each other (Spearman ρ = 0.415, *p* < 0.001). Of 190 study participants who replied to both HIV and substance use stigma questions, 20 (10.5%) had both HIV and substance use stigma scores above the 75th percentile, while 10.5% (n = 20) had only HIV stigma score above the 75th percentile and 14% (n = 26) had only substance use stigma score above the 75th percentile (Figure 1). The remaining 65% (n = 124) had medium or low scores (below the 75th percentile) for both forms of stigma.

### 3.5 Quality of care

The median HCQI score was 90 (IQR: 70‐90) out of 100 at co‐located OAT and ART sites and 80 (IQR: 60‐80) at OAT only sites (*p* < 0.001).

Compared to OAT only sites, patients at co‐located OAT and ART sites more frequently reported receipt of ART (90% vs. 74%, *p* = 0.005), undetectable viral load (59% vs. 41%, *p* = 0.012), receipt of isoniazid preventive treatment (77% vs. 46%, *p* < 0.001), and receipt of referrals for additional services or support (78% vs. 56%, *p* = 0.002). However, fewer reported receipt of HCV screening compared to patients at OAT only sites (91% vs. 99%, *p* = 0.011).

In adjusted ordinal regression, the quality of care score was associated with care co‐location (adjusted OR: 4.13; 95% CI: 2.31, 7.54), AUDIT‐C score (adjusted OR: 0.86; 95% CI: 0.76, 0.96), time since first HIV‐positive test result in years (adjusted OR: 1.08; 95% CI: 1.03, 1.13), and history of TB (adjusted OR: 2.93; 95% CI: 1.50, 5.87) (Table 3).

**Table 3:** Factors associated with perceived quality of HIV care^a^ among HIV‐positive PWID receiving OAT at seven sites in Ukraine: ordinal regression analysis, n = 191

	Crude OR (95% CI)	Adjusted OR (95% CI)
Co‐located OAT and ART sites (ref. OAT only sites)	**3.03 (1.78, 5.21)**	**4.13 (2.31, 7.54)**
Stigma		
HIV stigma score	1.01 (0.99, 1.03)	–
Substance use stigma score	0.99 (0.97, 1.01)	–
Dual stigma^b^ (ref. low/medium HIV and substance use stigma)		
High HIV and substance use stigma	1.05 (0.49, 2.28)	1.28 (0.57, 2.95)
High HIV stigma only	0.89 (0.42, 1.88)	0.65 (0.28, 1.44)
High substance use stigma only	**0.43 (0.19, 0.94)**	0.44 (0.18, 1.01)
Demographical characteristics		
Age, years	**1.06 (1.02, 1.10)**	1.04 (1.00, 1.08)
Male gender (ref. female)	1.00 (0.55, 1.82)	ref.
Married/have regular partner (ref. single, never married, divorced, separated or widowed)	1.01 (0.60, 1.70)	–
Household size, persons	0.94 (0.81, 1.10)	–
Living with children under 18 years (ref. no)	0.69 (0.39, 1.22)	–
High school or less education (ref. higher education)	1.36 (0.68, 2.73)	–
Vocational education (ref. higher education)	1.25 (0.63, 2.52)	–
Unemployed including housewives and students (ref. full or part‐time employment)	1.35 (0.80, 2.27)	–
Personal monthly income above the median: >1667 UAH^c^ (ref. median or below)	1.00 (1.00, 1.00)	–
History of incarceration (ref. no)	1.51 (0.88, 2.61)	–
Behavioral characteristics		
Any illicit drug use during the last 30 days (ref. no)	0.61 (0.36, 1.01)	–
1 sexual partner during the last 12 months (anal/vaginal sex) (ref. no partners)	1.16 (0.59, 2.24)	–
≥2 sexual partners during the last 12 months (anal/vaginal sex) (ref. no partners)	0.75 (0.32, 1.72)	–
Condomless sex during the last 12 months (anal/vaginal) (ref. no)	1.22 (0.72, 2.07)	–
History of selling sex (ref. no)	0.91 (0.31, 2.72)	–
Everyday or occasional smoker (ref. smoked before or never smoked)	0.36 (0.09, 1.22)	–
AUDIT‐C score	**0.84 (0.76, 0.94)**	**0.86 (0.76, 0.96)**
Clinical characteristics		
Time since first HIV‐positive test result, years	**1.08 (1.04, 1.13)**	**1.08 (1.03, 1.13)**
CD4 count	1.00 (1.00, 1.00)	ref.
History of TB (ref. no)	**2.94 (1.59, 5.55)**	**2.93 (1.50, 5.87)**
Diagnosed with HCV (ref. no)	1.85 (0.82, 4.20)	–
SF12v2 physical health T‐score	0.98 (0.95, 1.00)	–
SF12v2 mental health T‐score	1.01 (0.98, 1.04)	–
Ever attempted suicide (ref. no)^d^	**0.52 (0.29, 0.91)**	–
OAT drug: Methadone (ref. Buprenorphine)^d^	**0.71 (0.29, 0.91)**	–
OAT duration, years	1.11 (1.00, 1.23)	–
Attitudes		
Perceived accessibility of the healthcare facility	1.01 (1.00, 1.03)	–
Perceived user‐friendliness of the healthcare facility	1.01 (1.00, 1.03)	–
Social support score	1.00 (0.99, 1.01)	–
Receipt of services at non‐governmental organization (ref. no)	1.72 (0.97, 3.05)	–

Values in bold indicate statistically significant associations.

ART, antiretroviral treatment; AUDIT‐C, The Alcohol Use Disorders Identification Test, short version; CI, confidence interval; HCV, Hepatitis C Virus; OAT, opioid agonist therapy; OR, odds ratio; PWID, people who inject drugs; SF12v2, Short Form Health Survey questionnaire, version 2; TB, tuberculosis; UAH, Ukrainian Hryvnias.

^a^ HIV Care Quality Index (HCQI) was categorized by quartiles (<70, 70‐79, 80‐89, ≥90 out of 100 points).

^b^ High stigma score was defined as a score above the 75th percentile and low/medium stigma as a score below the 75th percentile.

^c^ 1U.S. dollar = 26 UAH.

^d^ Due to the limited sample size, history of suicide and type of OAT drug were not included in the multivariable model despite their significant association with HCQI in unadjusted analysis.

## 4 Discussion

In our study conducted at seven sites in Ukraine, receipt of co‐located HIV and OAT services was associated with better perceived quality of HIV care but was not linked to stigma levels. We found that regardless of the model of care (co‐located OAT and ART sites or OAT only services), one in ten people with HIV who injected drugs experienced high levels of both HIV and substance use stigma, which was associated with poorer mental health and less social support from NGOs. Intersection theory stipulates that multiple stigmas, rather than having additive effects, interact to create “intersectional” stigma [14]. Qualitative studies will be necessary to explore how both stigma forms intersect in this population, and to explain substance use stigma’s role in people who are also affected by HIV stigma.

While co‐located OAT and ART services care model might aim at reducing intersectional stigma related to HIV and substance use, other structural factors (e.g. social positions, processes, policies and environmental issues) may influence one’s stigmatization in the health care setting [39]. Thus, despite its potential to improve the HIV care cascade, care co‐location alone might not mitigate stigma. Unless privacy and confidentiality are protected for those who have not only addictions but also HIV, patients of co‐located OAT and ART sites may have concerns with their HIV status disclosure within the care setting [20, 21, 40]. In addition, other barriers such as concerns over HIV treatment efficacy, safety and tolerability might add to stigma [40].

According to this study's results, patients attending co‐located OAT and ART sites more frequently reported receipt of ART, uptake of isoniazid preventive TB treatment and receiving referrals for additional services, including psychosocial support. These findings on care quality are consistent with previous studies showing that co‐located HIV and addiction substance use treatment services improve accessibility of care, and therefore increase uptake of services [20, 21, 22].

In our study, three of four co‐located OAT and ART sites had also an NGO providing social support and harm reduction services for PWID, while the respective proportion was one in three at OAT only sites. In Ukraine, NGOs take an active role in advocating for user‐friendliness of services for key populations, such as attentiveness, respect and clarity of explanation in interaction between health providers and clients [41]. According to our findings, receipt of NGO services was associated with lower substance use stigma. However, it was not associated with better perceived quality of care.

There are several limitations to this study. The HIV and substance use stigma scales have not been validated in Ukraine, but we conducted factor analysis to assess internal consistency and validity. Given the cross‐sectional design, we cannot imply direction of the observed associations. We recruited participants at OAT sites, while participants’ stigma scores might primarily reflect previous experiences in other facilities and communities outside of current treatment settings. The accuracy of clinical information might have been limited by recall error. Finally, study results cannot be generalized to the entire population of HIV‐positive PWID in Ukraine, as only 6% of the estimated HIV‐positive PWID population receive OAT [29].

These limitations notwithstanding, this study’s results have several implications. First, treatment integration alone does not seem sufficient to address stigma among people with both substance use disorders and HIV. Stigma interventions, such as Acceptance and Commitment Therapy [42], targeted to HIV‐positive people with a history of substance use should therefore be designed and tested in both co‐located and not co‐located healthcare settings. Second, given the high intersectional stigma we found in this study, stigma interventions might benefit from incorporating an intersectionality framework to reduce shame and devaluation related to both substance use and HIV. Third, stigma’s association with suicide attempts and poor mental health might need particular attention. Stigma interventions need to consider mental health in their approach. Finally, NGOs with direct reach to stigmatized populations might be well suited to develop and implement stigma reduction interventions to empower affected people to cope effectively with stigma and its implications.

## 5 Conclusion

Patients receiving substance use treatment in Ukraine report high levels of both HIV and substance use stigma linked to poorer mental health and less social support by NGO. Co‐located treatment did not seem to provide a stigma‐free environment for this key population. Stigma interventions for HIV‐positive PWID, possibly delivered involving peers at NGOs, are required to help affected people cope with negative emotions and experiences associated with stigmatization.

## Competing Interests

The authors declare that they have no conflict of interest.

## Authors’ Contributions

YS and TK drafted the manuscript. YS created data collection tools and conducted the data analyses, with substantial contributions to the analytic plan from KL and TK. KL and TK conceived of and designed the study, with substantial contributions from JS and TF. OM and TK led the data acquisition, with substantial contributions from JJC, NR, AC, SB, BI, AD and FSG. OM, JJC, NR, AC, SB, BI, AD, FSG, JHS, TF and KL substantively revised the manuscript for important intellectual content.
